# Supplementary material for: Finding Species-Specific Extracellular Surface-Facing Proteomes in Toxic Dinoflagellates
Source: Toxins (Basel). 2021 Sep 6;13(9):624. doi: 10.3390/toxins13090624 (PMC8473415; doi:10.3390/toxins13090624)
Supplement: Supplementary file 1 [file toxins-13-00624-s001.zip › toxins-1336147-supplementary.pdf]

Supplementary Information

# Finding Species-Specific Extracellular Surface-Facing Proteomes in Toxic Dinoflagellates

**Kenrick Kai-yuen Chan [1], Hang-kin Kong [1,2], Sirius Pui-kam Tse [1], Zoe Chan [1], Pak-yeung Lo [1], Kevin W.H. Kwok [1,2] and Samuel Chun-lap Lo [1,2] ***

[1] Department of Applied Biology and Chemical Technology, Faculty of Applied Science and Textiles, The Hong Kong Polytechnic University, Hung Hom, Hong Kong; kai-yuen.ky.chan@connect.polyu.hk (K.K.C.); hang-kin.kong@polyu.edu.hk (H.K.); pktse@polyu.edu.hk (S.P.T.); zoe-z.c.chan@polyu.edu.hk (Z.C.); pypatlo@polyu.edu.hk (P.L.); kwh.kwok@polyu.edu.hk (K.W.H.K.)
[2] Research Institute for Future Food, The Hong Kong Polytechnic University, Hung Hom, Hong Kong
* Correspondence: Samuel.chun-lap.lo@polyu.edu.hk; Tel.: +852-34008669; Fax: +852-23649932

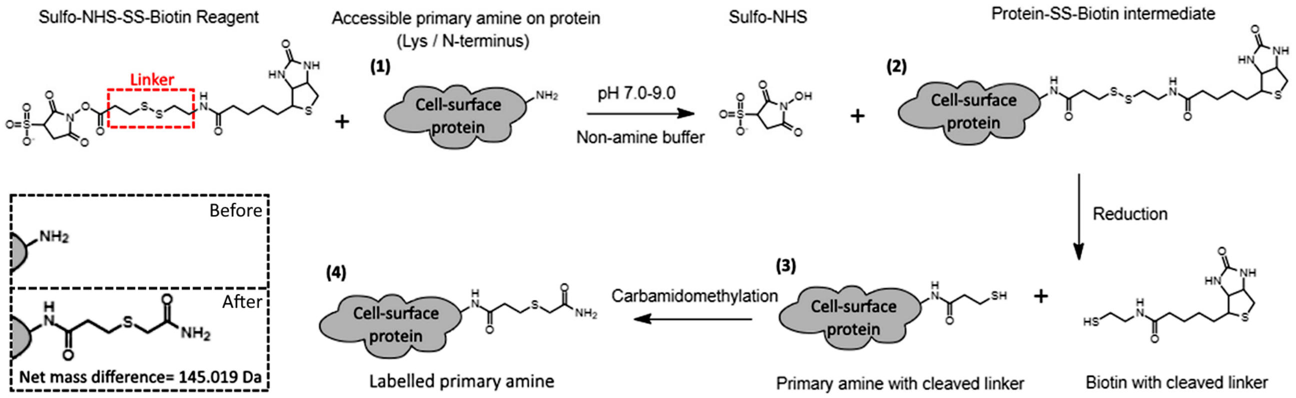


**Figure S1.** Reaction scheme of surface labeling using sulfo-NHS-SS-biotin reagent.

Four major reactions involved: (1) The exposed primary amines on the cell surface proteins reacted with the *N*-Hydroxysuccinimide (NHS) of the labeling reagent and yielded protein-SS-biotin intermediates. (2) The disulfide bond on the intermediate was reduced and disconnected by dithiothreitol (DTT) reduction during the mass spectrometry sample preparation procedure, and thus the side chain with biotin tag was removed. Primary amine was labeled partially with the leftover linker. Biotin with partial linker was removed during the C18 reverse phase chromatography. (3) The thiol group of the partially labelled primary amine was carbamidomethylated with iodoacetamide (IAA). (4) The completely labelled primary amine on a peptide would provide an increased mass difference of 145.019 Da.

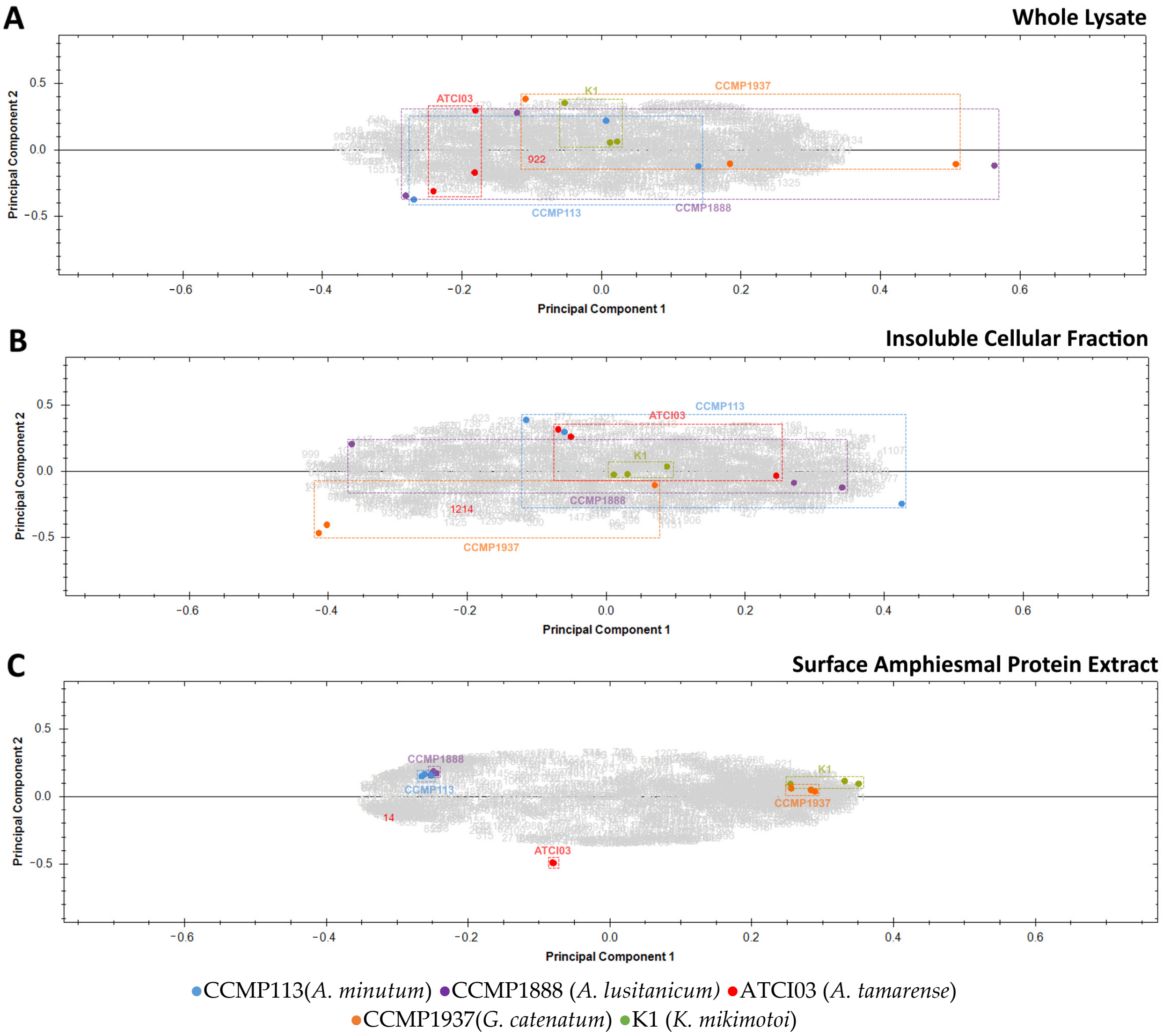


**Figure S2.** Difference in principal components of (**A**) whole lysates, (**B**) insoluble cellular fractions and (**C**) surface amphiesmal protein extracts of the five species studied.

Area of the boxes with dashed line indicated the consistence of the samples from same species. The larger of the box represented less consistence of the samples from the same species. Among three methods of extraction, novel amphiesmal extracts showed the best consistence and species coherence comparing to whole lysates and insoluble cellular fractions.

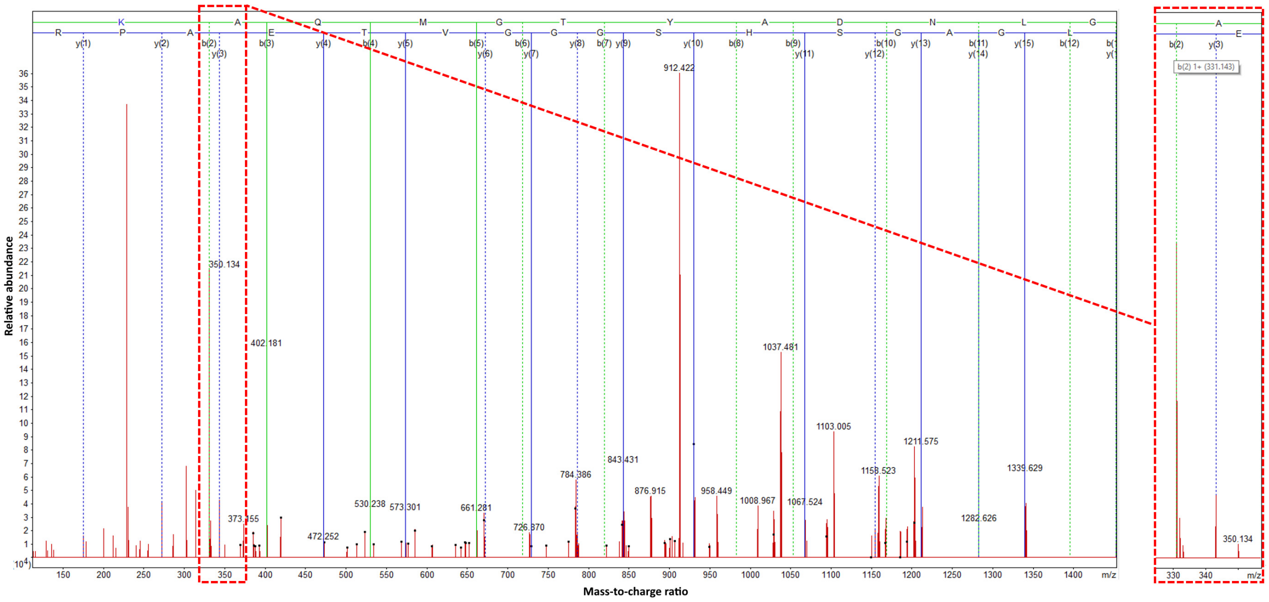


**Figure S3a**. MS/MS spectrum of a labeled peptide from a specific ortholog of K1.

The amino acid sequence of the peptide identified was "GKAQMGTYADNLGAGSHSGGGVTEAPR" and the actual mass of its b(2) ion was 331.143 Da. The tagged peptide was accurately identified by the LC-Orbitrap-MS that the difference between the actual and expected mass of the b(2) ion of labeled peptide was 0.00025 Da, which was within the mass tolerance and thus neglectable. Also, the modification was occurred on the expected primary amine containing lysine residue. This validated the applicability of this labeling reagent towards dinoflagellates. More importantly, mutual authentication from the annotation databases and the tag significantly indicated the confidence of the identified cell-surface proteins.

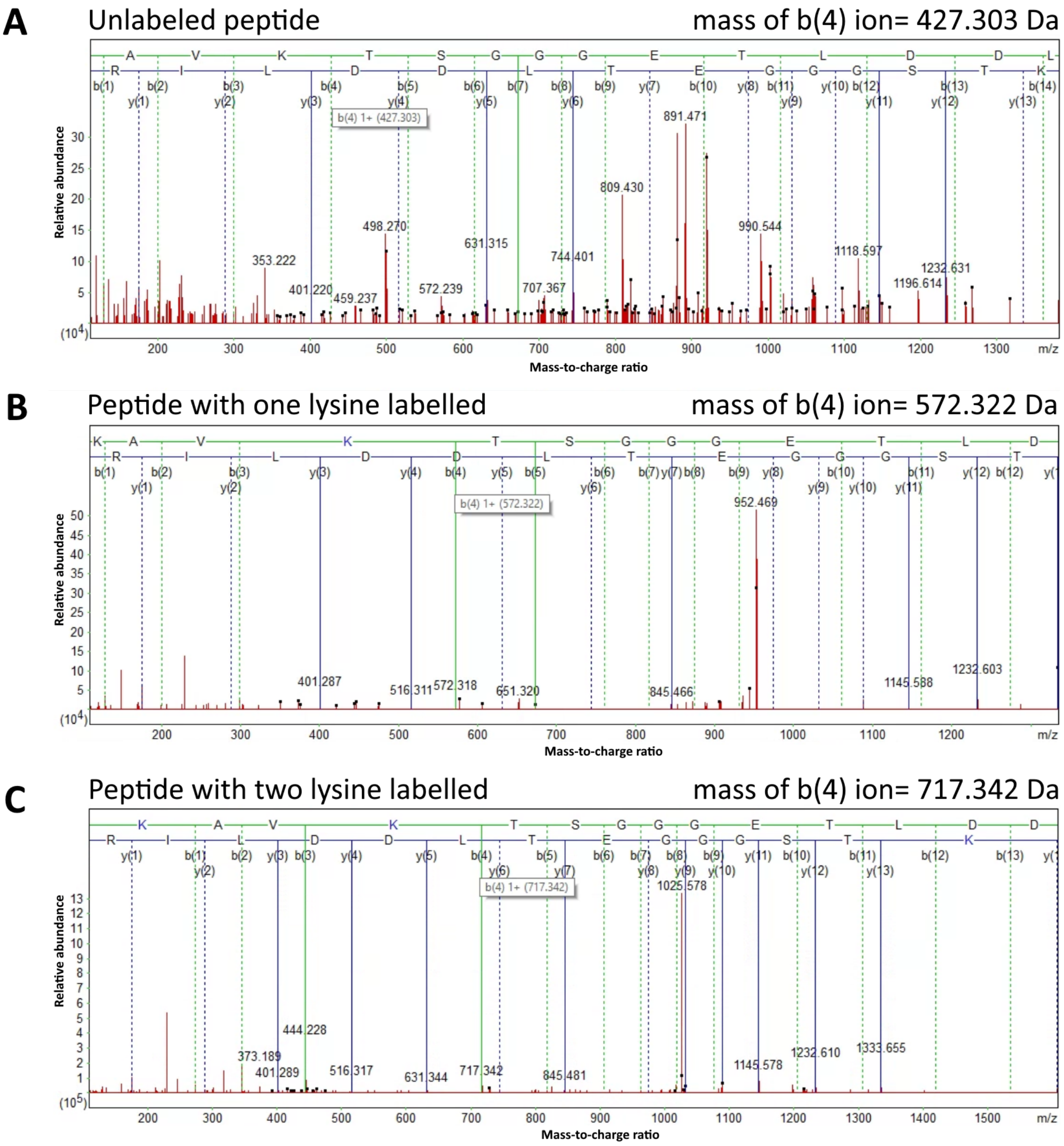


**Figure S3b**. Mass difference of the same peptide species identified in K1 with different numbers of lysine residue labelled: (**A**) Unlabeled peptide; (**B**) Peptide with one lysine residue labeled; (**C**) Peptide with two lysine residues labeled.

Another example of a peptide species identified in K1 with different numbers of labeled lysine residue. The amino acid sequence of the peptide species identified was "KAVKTSGGGETLDDLIR". This peptide contained two possible residues (lysine) being labelled. In the native unlabeled peptide, the actual mass of the b(4) ion was 427.303 Da. Increases of 145.019 Da and 290.039 Da were observed in the b(4) ion of the singly labeled peptide shown in (B) and doubly labeled peptide shown in (C) respectively.

**Table S1.** DNA sequences of the PCR products.

**ITS-DNA Sequence(s) of CCMP113**

>CCMP113_Pair1
TCTGCTCCACGCGTTGGGAGCTCTCCCATATGGTCGACCTGCAGGCGGCCGCGAATTCACTAGTGATTTCCTCCGCTTATTGATATG
CTTAAATTCAGCAGGTTTACATGCTTCACTTCATGTTAAGTTTACGCTGTTCAGCTACCCAGACAAATACTATGCAACCAATGCAAG
TGACAAAGGCTAGACCTAAGCAAACACAATGAGGTTGCAATGCATTCGCAAGCGCGGGTGTCCAATGCAACAGCATTGACACACACA
GCTCACAACGTTGCTGAACAGCAAATTCAATGTTGGAAGTTAAGTGACATTGAATCAAGCACACCTTCAAGCATATCCCGAAGGTGC
AAATTACGTTCAAACATCTATTGGCTCACGGAATTCTGCAATTCACAATGCATATCACATTTTGCTGCATTCTTCATCATCAATTGA
GCTAAGACATTCATTGCAAAAACACATTTCACATTCAAGTTCAATGCAAGAAACAAGTACAAGCAATCAACAAAAGGTCATCAACAC
AGCAGCATGAAAGCGCTTGCAGAAGCAAGCCATGCCACCCACGAGCAAATGATGCAGAACCTAAAGCCTAGGAACCCCACCACAGCT
CACAAAGTCGTGAAAGATTAATGTTGTTAGGAATGTGCAAATGATCCTTCCGCAGGTTCACCTACGGAAATCGAATTCCCGCGGCCG
CCATGGCGGCCGGGAGCATGCGACGTCGGGCCCAATTCGCCCTATAGTGAGTCGTATTACAATTCACTGGCCGTCGTTTTACAACGT
CGTGACTGGGAAAACCCTGGCGTTACCCAACTTAATCGCCTTGCAGCACATCCCCCTTTCGCCAGCTGGCGTAATAGCGAAGAGGCC
CGCACCGATCGCCCTTCCCAACAGTTGCGCAGCCTGAATGGCGAATGGACGCGCCCTGTAGCGGCGCATTAAGCGCGGCGGGTGTGG
TGGTTACGCGCAGCGTGACCGCTACACTTGCCAGCGCCCTAGCGCCCGCTCTTTCGCTTTCTTCCCTTCCTTTCTCGCACGTTCGCG
CTTTCCCCGTCAGCTCTAATCGGGGCTCCTTAGGTTCGATTAGTGCTTACGCACTCGACCCCAAAACTTGATAGTGATGATCACGTA
ATGGCAATCGCCTGATAGACGGATTTTCGCCTGACTGGTCCACGTCTTAATATGACTCTTGTTCCAACCTGG

>CCMP113_Pair2
ACCAAGTCGCATGCTCCCGGCCGCCATGGCGGCCGCGGGAATTCGATTAGAACCTTACCACTTAGAGGAAGGAGAAGTCGTAACAAG
GTTTCTGTAGGTGAACCTGCGGAAGGATCATTTGCACATCCCTAACAACATTAATCTTTCACGACTTTGTGAGCTGTGGTGGGGTTC
CTAGGCTTTAGGTTCTGCATCATTTGCTCGTGGGTGGCATGGCTTGCTTCTGCAAGCGCTTTCATGCTGCTGTGTTGATGACCTTTT
GTTGATTGCTTGTACTTGTTTCTTGCATTGAACTTGAATGTGAAATGTGTTTTTGCAATGAATGTCTTAGCTCAATTGATGATGAAG
AATGCAGCAAAATGTGATATGCATTGTGAATTGCAGAATTCCGTGAGCCAATAGATGTTTGAACGTAATTTGCACCTTCGGGATATG
CTTGAAGGTGTGCTTGATTCAATGTCAATTAACTTCCAACATTGAATTTGCTGTTCAGCAACGTTGTGAGCTGTGTGTGTCAATGCT
GTTGCATTGGACACCCGCGCTTGCGAATGCATTGCAACCTCATTGTGTTTGCTTAGGTCTAGCCTTTGTCACTTGCATTGGTTGCAT
AGTATTTGTCTGGGTAGCTGAACAGCGTAAACTTAACATGAAGTGAAGCATGTAAACCTGCTGAATTTAAGCATATAAGTAGGCGGT
GGAAAATGAACCAAATGGGATTCCTTAAGTAATTGCGAATGAAGAGGGATTTGCTTAGCAATCACTAGTGAATTCGCGGCCGCCTGC
AGGTCGACCATATGGGAGAGCTCCCAACGCGTTGGATGCATAGCTTGAGTATTCTATAGTGTCACCTAAATAGCTTGGCGTAATCAT
GGTCATAGCTGTTTCCTGTGTGAAATTGTTATCCGCTCACATTTCCACACAACATACGAGCCGGAAGCATAAAGTGTAAAGCCTGGG
GTGCCTAATGAGTGAGCTAACTCACATTAATTGCGTTGCGCTCACTGCCCGCTTTCCAGTCGGGAAACCTGTCGTGCCAGCTGCATT
AATGAATCGGCCACGCGCGGGGAGGAGCGGTTTGCGTATTGGGCGCTCTTCGCTTTCTCGCTCACTGACTCGCTGCGCTCGTCGTTC
GCTGCGGCGAGGCGTATCAGCTCACTCAAAGGCGGTATTCGCTTATCCAAGGATTCAGGGATACGCAGGAAGACTGGTGGAGCAGGC
CAGCTAGCTCAGGACTCGTAAAAGGGCCCCGCTGTGTCTGTTGCGT

**ITS-DNA Sequence(s) of CCMP1888**

>CCMP1888_Pair1
CTCAGCTCCCGGCCGCCATGGCGGCCGCGGGAATTCGATTTCCTCCGCTTATTGATATGCTTAAATTCAGCAGGTTTACATGCTTCA
CTTCATGTTAAGTTTACGCTGTTCAGCTACCCAGACAAATACTATGCAACCAATGCAAGTGACAAAGGCTAGACCTAAGCAAACACA
ATGAGGTTGCAATGCATTCGCAAGCGCGGGTGTCCAATGCAACAGCATTGACACACACAGCTCACAACGTTGCTGAACAGCAAATTC
AATGTTGGAAGTTAATTGACATTGAATCAAGCACACCTTCAAGCATATCCCGAAGGTGCAAATTACGTTCAAACATCTATTGGCTCA
CGGAATTCTGCAATTCACAATGCATATCACATTTTGCTGCATTCTTCATCATCAATTGAGCTAAGACATTCATTGCAAAAACACATT
TCACATTCAAGTTCAATGCAAGAAACAAGTACAAGCAATCAACAAAAGGTCATCAACACAGCAGCATGAAAGCGCTTGCAGAAGCAA
GCCATGCCACCCACGAGCAAATGATGCAGAACCTAAAGCCTAGGAACCCCACCACAGCTCACAAAGTCGTGAAAGATTAATGTTGTT
AGGAATGTGCAAATGATCCTTCCGCAGGTTCACCTACGGAAATCACTAGTGAATTCGCGGCCGCCTGCAGGTCGACCATATGGGAGA
GCTCCCAACGCGTTGGATGCATAGCTTGAGTATTCTATAGTGTCACCTAAATAGCTTGGCGTAATCATGGTCATAGCTGTTTCCTGT
GTGAAATTGTTATCCGCTCACAATTCCACACAACATACGAGCCGGAAGCATAAAGTGTAAAGCCTGGGGTGCCTAATGAGTGAGCTA
ACTCACATTAATTGCGTTGCGCTCACTGCCCGCTTTCCAGTCGGGAAACCTGTCGTGCCAGCTGCATTAATGAATCGGCCAACGCGC
GGGGAGAGGCGGTTTGCGTATTGGGCGCTCTTCGCTTCTCGCTCACTGACTCGCTGCGCTCGGTCGTTCGGCTGCGCGAGCGGTATC
AGCTCACTCAAGCGTATACGTTATCCACAGAATCAGGGATACGCAGAAAGAACATGGTGGAGCAAAGGCAGCAAGTAGATCGTAAAA
GGCCCGCGATGCTGGCGTTTCCCTAGGTTCGCCTCCTTGACCAGACTTCGAATTCGCACGCCTCAAGTCCTAAGGGTGGTGC

**ITS-DNA Sequence(s) of ATCI03**

>ATCI03_Pair2
AGTCATCCAACGCGTTGGGAGCTCTCCCATATGGTCGACCTGCAGGCGGCCGCGAATTCACTAGTGATTGCTAAGCAAATCCCTCTT
CATTCGCAATTACTGAAGAGATCCCAGTTGGTTTAATTTCCACCACTTACTTATATGCTTAAATTCAGCAGGTTTATGTGCTTCACT
TCACGCTTAGCATATGCAGGTCTTCAGCTTGTCCCAGTTGTGCACATTTACAAATATCAACCATCAAATGACGAAACAGGTCTGCCC
CACGAAACCATGTTTAGGTTGTAAACAACTGTATGACATCCAGGTTCAATGCAACACATTGACCTTTATTGTTCACAACAATGCTAA
TCAGCATTAACTGTATATGAAAGCTACTTTGCATTAAACCAAGCAAACCTTCAAGAATATCCCAAAGGTACAAGTAACATTCAAACA
TCTGTTAGCTCACGGAATTCTGCAATTCGCAATGCATATTGCATGTTGCTGCATTCTTCATCATTTATTGAGCTAAGACATTCCTTG
CAAAACATAAGTGTTGCAGCAACTGGAGAAATGCAAACAGTTTACACAACACATCACACGCTACAGCACACATGCTTGAAAAACAAA
TCATGCTGCCCAGGGCAAGTGCAAGTCCATTGCAATACATGCTTACAGTTGCCCACAATATCATTAGCATGCAGTTGGCTACACATG
TGCAAATGATCCTTCTGCAGGTTCACCTACAGAAACCTTGTTACGACTTCTCCTTCCTCTAAGTGGTAAGGTTCAAATCGAATTCCC
GCGGCCGCCATGGCGGCCGGGAGCATGCGACGTCGGGCCCAATTCGCCCTATAGTGAGTCGTATTACAATTCACTGGCCGTCGTTTT

ACAACGTCGTGACTGGGAAAACCCTGGCGTTACCCAACTTAATCGCCTTGCAGCACATCCCCCTTTCGCCAGCTGGCGTAATAGCGA
AGAGGCCGCACCGATCGCCCTTCCCAACAGTTGCGCAGCCTGAATGGCGAATGGACGCGCCCTGTAGCGGCGCATTAAGCGCGGCGG
GTGTGGTGGTTACGCGCAGCGTGACCGCTACACTTGCCAGCGCCCTAGCGCCCGCCTCCTTTCGCTTTCTTCCCTTCCTTTCTCGCA

**ITS-DNA Sequence(s) of CCMP1937**

>CCMP1937_Pair1
AAAGGCCATCCAACGCGTTGGGAGCTCTCCCATATGGTCGACCTGCAGGCGGCCGCGAATTCACTAGTGATTTCCTCCGCTTATTGA
TATGCTTAAATTCAGCGGGTTTGCCAACCTAACTTCATGTCTTAAAGCTGGGCGCAGCCACAGTTGATCGATGCGAATGAAACATTG
ACACGTCGTGCGCAACTAGTTGATTCGCTTCAATCAGTTGGCAGCGTGACAACACCTTGTCAATCGAATGCCGCAAGAGCGCCACAC
ACCCTCTCGTCAGAGGACGCGCGTCTTGAAGCACGCATAATCACAGCAGAACGCACCTTCGAGAGGAAGAATGACAACTGCTGGAAA
TAACAACATAGACACTGAAGCAGGCATGCTTTCAGGGATATCCCAAAAGCACAATATGCGTTCAAGTTTCTGTCGGTTCACGGAATT
CTGCAATTCACAATGCTTATCGCACTTCGCTGCGCCCTTCATCGTTGTTCGAACCGAGACATCCGTCGCTGAAAGTTGTAATTGCTG
GTTGATACCAATGCAACGAATACAGCTTCACATTAGCCTGAGAGACTTCAGGTAGCAGGCACAAGGCGATTAAAGCACCGCCTTGCC
CCTGCTACCCTCGAGATTGTTCGTGGCGGCAACTAGAGGAGCAACTTCCACGTACAGCCTCACAGAAGAGTTCACAAGAATGGTTTT
GGATACGTGCGAATGATCCTTCCGCAGGTTCACCTACGGAAATCGAATTCCCGCGGCCGCCATGGCGGCCGGGAGCATGCGACGTCG
GGCCCAATTCGCCCTATAGTGAGTCGTATTACAATTCACTGGCCGTCGTTTTACAACGTCGTGACTGGGAAAACCCTGGCGTTACCC
AACTTAATCGCCTTGCAGCACATCCCCCTTTCGCCAGCTGGCGTAATAGCGAAGAGGCCCGCACCGATCGCCCTTCCCAACAGTTGC
GCAGCCTGAATGGCGAATGGACGCGCCCTGTAGCGGCGCATTAAGCGCGGCGGGTGTGTGGTTACGCGCAGCGTGACCGCTACACTT
GCCAGCGCCCTAGCGCCGCTCCTTTCGCTTTCTTC

**ITS-DNA Sequence(s) of K1**

>K1_Pair1
AAGCTCCACGCGTTGGGAGCTCTCCCATATGGTCGACCTGCAGGCGGCCGCGAATTCACTAGTGATTTGAACCTTATCACTTAGAGG
AAGGAGAAGTCGTAACAAGGTTTCCGTAGGTGAACCTGCGGAAGGATCATTCACACGCATCCAACTTGAATTCATTGTGAACCATCT
TCTGTGAGTTGTGCTCTTGGCTGGAGACAACTTGTCTACAACCTTGTGCAGAATCTCGCGGGTAGTTCGGGTGATATTGGACCGTCT
TCCTCTATTGTCTGGCTACCATTGTTGTCTGACCAATGTCTTGCATGATGACCTATCTTTTTAAGATGTGGGTGACCACATGTTTGA
TTCACATACAACTTTCAGCGACGGATGTCTCGGCTCGAACAACGATGAAGGGCGCAGCGAAGTGTGATAAGCATTGTGAATTGCAGA
ATTCCGTGAACCAATAGGGATTTGAACGTATACTGCGCTTTCGGGATATCCCTGAAAGCATGCCTGCTTCAGTGTCAATATTCTCTC
ATGCCACTGTCATCTTGTCATGTGCTTTGCACATGCAACTGACAGCAGTGTGTGTCTGTGCATTAAGGTGCTCTTCCTGCCCCTGAT
GCATTGAATCCATGGGTTTTGTGCTCTGTCAGCAACTTGATAAACACCTTGTGCTTTGTGTGTAACCTGTTGCTTTGTCTGTTGCAC
TGATAACCTGTCCTCCTGTCTGCCACTTCATTTGTGGCTTCCTTCCATGACATGAAGTTAGGTAAGCAAACCCGCTGAATTTAAGCA
TATAAGTAAGCGGAGGATAAGAAACTAAATAGGATTCCCTCAGTAATGGCGAATGAACAGGGATTAGCTCAGCAATCGAATTCCCGC
GGCCGCCATGGCGGCCGGGAGCATGCGACGTCGGGCCCAATTCGCCCTATAGTGAGTCGTATTACATTTCACTGGCCGTCGTTTTAC
ACGTCGTGACTGGGAAAACCCTGGCGTTACCCAACTTAATCGCCTTGCAGCACATCCCCCTTTCGCCAGCTGGCGTAATAGCGAAGA
AGGCCCGCACCGATCGCCCTTCCCAACAGTTGCGCAGCCTGAATGGGCGAATGGGAACGCGGCCCTGTTAACGGGCCGCCATTATAC
CCCGCCGGGGGGGGTGGTGGGG

>K1_Pair2
TTTCTCCAACGCGTTGGGAAGCTCTCCCATATGGTCGACCTGCAGGCGGCCGCGAATTCACTAGTGATTTCCGTAGGTGAACCTGCG
GAAGGATCATTCACACGCATCCAACTTGAATTCATTGTGAACCATCTTCTGTGAGTTGTGCTCTTGGCTGGAGACAACTTGTCTACA
ACCTTGTGCAGAATCTCGCGGGTAGTTCGGGTGATATTGGACCGTCTTCCTCTATTGTCTGGCTACCATTGTTGTCTGACCAATGTC
TTGCATGATGACCTATCTTTTTAAGATGTGGGTGACCACATGTTTGATTCACATACAACTTTCAGCGACGGATGTCTCGGCTCGAAC
AACGATGAAGGGCGCAGCGAAGTGTGATAAGCATTGTGAATTGCAGAATTCCGTGAACCAATAGGGATTTGAACGTATACTGCGCTT
TCGGGATATCCCTGAAAGCATGCCTGCTTCAGTGTCAATATTCTCTCATGCCACTGTCATCTTGTCATGTGCTTTGCACATGCAACT
GACAGCAGTGTGTGTCTGTGCATTAAGGTGCTCTTCCTGCCCCTGATGCATTGAATCCATGGGTTTTGTGCTCTGTCAGCAACTTGA
TAAACACCTTGTGCTTTGTGTGTAACCTGTTGCTTTGTCTGTTGCACTGATAACCTGTCCTCCTGTCTGCCACTTCATTTGTGGCTT
CCTTCCATGACATGAAGTTAGGTAAGCAAACCCGCTGAATTTAAGCATATCAATAAGCGGAGGTAATCGAATTCCCGCGGCCGCCAT
GGCGGCCGGGAGCATGCGACGTCGGGCCCAATTCGCCCTATAGTGAGTCGTATTACAATTCACTGGCCGTCGTTTTACAACGTCGTG
ACTGGGAAAACCCTGGCGTTACCCAACTTAATCGCCTTGCAGCACATCCCCCTTTCGCCAGCTGGCGTAATAGCGAAGAGGCCCGCA
CCGATCGCCCTTTCCCAACAGTTGCGCAGCCTGAATGGCGAATGACGCGCCCTGTAGCGCGCATTAAGCGCGGCGGGGTGTGGTGTA
CGCGCAGCGTGACCGCTACACTGCAGCGCCTACGCCCGCCTCCTTCGCTTTCCTGATGCTTCTCGCCAAGTCGCGGCTTCCGTCAGC
TCTAATCGGGGGCTCCGTTAGGTCGAATTATGTCTTTACGACCTGACCGAAACTGGATAGGTAGGTCACCTATGTGG

**Table S2.** Statistics of proteins identified in whole lysate (WL) and insoluble cellular fraction (ICF).

| No. of Protein Identified | Dinoflagellate Species | | | | |
|---|---|---|---|---|---|
| | CCMP113 | CCMP1888 | ATCI03 | CCMP1937 | K1 |
| ICF | | | | | |
| Amphiesmal | 356 | 506 | 439 | 523 | 414 |
| Non-Amphiesmal | 524 | 781 | 687 | 726 | 615 |
| Unknown | 352 | 590 | 408 | 571 | 379 |
| Total | 1,232 | 1,877 | 1,534 | 1,820 | 1,408 |
| WL | | | | | |
| Amphiesmal | 393 | 371 | 451 | 422 | 378 |
| Non-Amphiesmal | 743 | 583 | 813 | 678 | 620 |
| Unknown | 613 | 475 | 550 | 557 | 506 |
| Total | 1,749 | 1,429 | 1,814 | 1,657 | 1,504 |

**Table S3.** Uniprot annotations and amino acid sequences of transporter homologs identified.

**Transport Protein(s) Identified in CCMP113**

**Best-matched Uniprot annotation of homologs identified (Accession numbers):**
**Clathrin heavy chain 1 (P499510, Q2RBN7)**

**(Mascot score: 502)**

```
>Cluster-16584.55282
MMQAPMMAAGAQAAPAEPPISLSTTLNLADHGINAQAFRFGNLTMESDRWLSVKDAAADG
SGQVVVIDMHNGNAVNRRPMKAEASLMNPNDNIMALKGSTEGQPGHFVQVFNLDTKEKLG
VYQAPENLVFWRWLSPRMLALVGEKNVYHWNLEVANSTPETVFQRSGKLAEAGSQVISYA
ANSQLSWCLLTAISTQDQGRTIDGNMQLYSVEKKQQQMLEGHAGCFGNITVADGEGPAGL
LAFMERKAGSLQTKLHVMDVTKPRGEGLPPPFKMQSEVAMPPEAPNDFAVALHLSEKHGV
AFMVTKAGYLFVFDIATATMLVRTRVSQDTIFISTYSSLSGGCIFVNRKGAVLTAKVNEP
TIVSYIMNSLVQLSNRQDVAFNLAKRFGLPGADELFQRQFSHYFASGDYKNAALVAAQCK
SGALRSPQTIQQFKSVQAPAGQSSPILHYFSTLLEYGRLNALESVELARPVVQQQRRELV
EKWLKEDKLECSEELGDIVRPLETKFALSIYLRANAHQKAIAAFVELGQYDQVAAYARKV
GYQADYSSLLQQMLSTSPEGATNFAKSLLTGQSGGPPLIDINQVVKVFMDQNRLQETTSI
LLEALKENRPDQAQLQTQLLAMNLQQAPKVAEAIMQMNMFTHYDRHYIGQLCEKAGLMQR
ALEHYQDSADLKRVMLHAHQMSPEFLTQYFSRMPPDTALECLYDLLRHNRQNLNVAVQVA
IKYHEQIGAAKIVEMFESFGSNEGVFYFLGAILSSSTDPLVHYKYIQAASRCGNMQEVER
VCRESTCYDPATVKDFLKDTKLPDPRPLIYVCDLHGFVGELTEYLYKNSLMKYIEVYVVK
VNPTNCPTVIGTLIDLDCSEDFIKTLLQNVRAACPIEPLVAEVEKRNRLRVLLPWLEARV
AEGNQDPYLHNGIAKIYIDTNRDPETFLKNNAFYDSATVGKYCEDKDPHLAFTAYKRAWG
SCDQQLVDVTNRNLLWRLQARYLVERQSPELWATVLDPENQHRRSVIDMVVSTALPEAKD
ADEVSATVRAFISADLPNELIELLEKIVLHNSNFSKNKNLQNLLILTAIKADKSRVMDYI
NRLDNYDGPDIAKIALGDPYHLYEEAFLIYKKCGQNAEAMETLLENIESLERAQEFAARC
NEGPVWYRLGKAQLENGAVPEAIESYLRAEDASDYQQVIQVAEREENYEELVRFLVMARS
TAKDQLVDTELVYAYAKTDRLAEMEEFVTGTNTANVQQVGDRLFEERAHKAAKLLYQSIP
NNAKLASCHVQLGEFTQAVDAARKANNPRTWKEVNMACVQAQQFRCAEIAGMHIIVHPDH
LEELITQYEAGGHFEELIALLDSGLTSDRAHVGMYTELAVLYAKYRSEKLMDFIKLNTAR
LNIPKLIHACERHHLWQHVIFLYTHYDEFDSAANTMMAHSPTAFAHDQFQMIMQKVSNTE
LYYRAVQFYLEEQPMQINSLLTTVTPKVDHARVVQQVRKSGHLSLIMPYLKAVQQHNLQP
VNEALNELYVEAEQHEELRGSIEDFDNIDQIGLAQKLERHELVEMRRIAALVYKKNKRYK
QSIDLSKLDRMYKDAMETARDSGNNDLAESLLRYFVDEDMRECFAACLFTCYELIRPDVG
VELAWRKGMLDFAMPFLIQVLREYTGRIDALDKKTQKKEEAEEKQKSAPNDYVPDYIGPP
MMGPGGLAGFGGLALTSGPAMPQVQQPGFPQPSMMQAGPGMMMMPPGAGGMR
```

**(Mascot score: 232)**

```
>Cluster-16584.53449
MGGPQPPTPPARWLRAQPPPPPRDTRPKPRAMMQAPMMAAGAQAAPAEPPISLSTTLNLA
DHGINAQAFRFGNLTMESDRWLSVKDAAADGSGQVVVIDMHNGNAVNRRPMKAEASLMNP
NDNIMALKGSTEGQPGHFVQVFNLDTKEKLGVYQAPENLVFWRWLSPRMLALVGEKNVYH
WNLEVANSTPETVFQRSGKLAEAGSQVISYAANSQLSWCLLTAISTQDQGRTIDGNMQLY
SVEKKQQQMLEGHAGCFGNITVADGEGPAGLLAFMERKAGSLQTKLHVMDVTKPRGEGLP
PPFKMQSEVAMPPEAPNDFAVALHLSEKHGVAFMVTKAGYLFVFDIATATMLVRTRVSQD
```

TIFISTYSSLSGGCIFVNRKGAVLTAKVNEPTIVSYIMNSLVQLSNRQDVAFNLAKRFGL
PGADELFQRQFSHYFASGDYKNAALVAAQCKSGALRSPQTIQQFKSVQAPAGQSSPILHY
FSTLLEYGRLNALESVELARPVVQQQRRELVEKWLKEDKLECSEELGDIVRPLETKFALS
IYLRANAHQKAIAAFVELGQYDQVAAYARKVGYQADYSSLLQQMLSTSPEGATNFAKSLL
TGQSGGPPLIDINQVVKVFMDQNRLQETTSILLEALKENRPDQAQLQTQLLAMNLQQAPK
VAEAIMQMNMFTHYDHQYIGQLCEKAGLMSWALEHYQDKADVKRAMLQAHLMTPEALIRF
FGRLPPETSLECMYDLMRHNRQNLNVVVQVAIKYNEQIGVMKIVEMFESFASNEGIFYLL
GGILSTCTDPDVHFKYIQAASRCGNMQEVERVCRESSVYDPLKVKEFLKEAKLPDPRPLI
YVCDLHNFVDELTEYLYKNSLMKYIEVYVVKVNPVKCPTVVGALVDMDCSEDFIKSLLQN
VRNACPIEPLVAEMEKRNRLRVLLPWLEQRVAEGNQDPALHNALAKICIDTNKDPENFLK
TNAFYDSATVGKYCEERDPHLAYTAYKRAWGSCDNQLVDVTNRNCLWRLQAQYLVGRQSP
DLWALVLNEDNKYRRSVIDQVVSTALPECTSADEVSSTVKAFISADLPNELIELLEKIVF
HKSADFSKNKNLQNLLILTAVKADTTRVMDYINRLDNYDGPEIARIALGEPYCLTEEAFV
IYKKCEMNSEAMDVLLNNIGSMERAQEFAARCNDSEVWYKLGRAQLGGGQVAETIEAHLK
AENANDFAQVIQAAEAQGLYDQLVQYLLMARTKAKDQLIDTELVYSYAKSDRLADMEEFV
GGTNTANIQSVGDRLYEEESYKAAKILFQSISNSAKLASCHVQLGEFTQAVDAAKKASNP
KTWREVNIACVQAQQFRCAEIAAMYIIVHPDHLEELITQYEAGGHFEELIALLDSGLTSD
RAHVGMYTELAVLYAKYRSEKLMDFIKLNTARLNIPKLIHACERHHLWQHVIFLYTHYDE
FDSAANTMMAHSPTAFAHDQFQMIMQKVSNTELYYRAVQFYLEEQPMQINSLLTTVTPKV
DHARVVQQVRKSGHLSLIMPYLKAVQQHNLQPVNEALNELYVEAEQHEELRGSIEDFDNI
DQIGLAQKLERHELVEMRRIAALVYKKNKRYKQSIDLSKLDRMYKDAMETARDSGNNDLA
ESLLRYFVDEDMRECFAACLFTCYELIRPDVGVELAWRKGMLDFAMPFLIQVLREYTGRI
DALDKKTQKKEEAEEKQKSAPNDYVPDYIGPPMMGPGGLAGFGGLALTSGPAMPQVQQPG
FPQPSMMQAGPGMMMMPPGAGGMR

**Best-matched Uniprot annotation of homologs identified (Accession numbers):**
**NAD(P) transhydrogenase, mitochondrial (Q61941)**

**(Mascot score: 392)**

>Cluster-16584.47837
MRAVVSGALRRGARQRRLFSDVRVGVLKESAGEQRVALVPSVAQKLIKDGYAVEVESGAGA
VAGFADQQYKDAGCAIATRNDVIKNSEMLFSVNPPPIGELDSMKGKTAVSWVGRRLPDAKE
LLTKAASSGVQLVDLTAVPRITIAQKLDVLSSQAKCAGHRAVMEAAHVYGRFFAPEVTAAG
KYPPTNTMILGVGVAGLAAIGTSKALGSVVRAWDVRDVSDQVQSMGASWITVDFEESGAGE
GGYAKESSAEFQQAQKDTFHKHLKECEIVITTAAIPGRPSPVLIEEYMVKDMKPGSVIVDL
AAVGGGNCKLTRKDEAFTTENGVTIIGYTDLAGRMAAQSSAMFAQNLANLLGHVSPKEKAK
GFFPSIDAAMTAGEDGDIVVRSIICTRDGAEVAMPPPPQPTPPKPKAAAVAAAAPKEVSIF
RQDMTNALLVTGGNAIMLGMGTAVPSHLLGTFTLAGAAGYQVVWGVKHALHTPLMSVTNAI
SGATAFGGLMLLEHTSGLAYCLAGLATTASAVNIFGGFIVTQRMLDLFKRPGDEDYSALYA
IPGAALVAAPLAGLATVESTGVVSQLLCIGAIGGLSSMTTAQMGCKLGMIGMAGGLTTTML
GMPASTYPAAAGMLASGAAMGTALGMKVEPIALPQTVAAFHSLVGLAAMITSVASHQAHPV
PGANLHNVACIVGDYIGGVTLAGSLVAFGKLNGNLGSAPLSLPGKNLLNAAGFAAQVGLGA
TFLTAGGELPLWATVALSNAMGYHLVASVGGADMPVCITVLNSYSGWALVAEGFLLDSPLV
TVIGSLIGFSGAILTKIMCDAMNRDIMNVIFGGINVAPKKEGETVQKEHTETNADGAAAAL
AESQSVMIVPGYGMAVARCQNSVAAIAQTLVNKGVKVRFGIHPVAGRMPGQMNVLLAEAGV
PYDWVQEMEEVNPDMDSVDTCLIVGANDTTNSGAQEGDPDHPLAGMPVIEVWKAKKVIFMK
RTMGAGYADVENPVFYKENTDMLLGNATDTTEQIAAKVKELVG

**Best-matched Uniprot annotation of homologs identified (Accession numbers):**
**ATP synthase subunit alpha, mitochondrial (P80021)**

**(Mascot score: 2,254)**

>Cluster-16584.49485
MCSPPSPPRQTMASRALLRRLVAGGARGALAPAAQTLAAAPAASRPLHTSAAALAAKVSP
AELTKILSERIANFQDQQDVQEVGRVLSVGDGIARIYGLKGVKAGEMVEFSSGLKGMALN
LETDNVGVVVFGDDRAIVEGDSVKCTGTIVDVPIGDGLLGRVVDALGTPIDGAGPIQTKE
RRRVELKAPGIIPRQSVHEPMTTGLKAVDSLIPVGRGQRELIIGDRQTGKTAIAIDTILN
QRAVNASADTNSHLYCIYVAVGQKRSTVAQLFEILRKNDCLKYTTIVAATASDAAPLQFL
APYTGCAMAEYFRDNGKHAVIFYDDLSKQAVAYRQMSLLLRRPPGREAYPGDVFYLHSRL
LERAAKMNEETHGGGSLTALPVIETQAGDVSAYIPTNVISITDGQIFLETELFYKGIRPA
VNVGLSVSRVGSAAQIKAMKQVAGTLKLELAQYREVAAFAQFGSDLDASTQHQLLRGGIL
TELLKQKQFVPMKSQEIIVSLWSGTRGYLDKVATKEILRFEALWLEHIKSTRAALLEDIM
AKKQLTTEIESGLKSAMEEFLGANEFEGRSS

Peptide ion identified in Mass Spectrometry

Amino acid residue labeled with isotopic tag

**Table S4.** Uniprot annotations and amino acid sequences of species-specific surface orthologs identified.

**Ortholog(s) Specific to CCMP113**

**Orthogroup number: OG0045624**
**Best-matched Uniprot annotation of orthologs (Accession numbers):**
**Isocitrate dehydrogenase [NADP] (P16100)**

**(Mascot score: 112; Peptide score: 60.3)**

```
>Cluster-16584.46230
MDIGGYYRLDKAKADAAMNPSATLNTIIANLEGAKKATGSSGAKKATKITYTYTDEAPML
ATHAFYPIIQSFCHHAGVPVELKDISVAGRVIAHFPECLTEEQRTGDILSELGLLAQQGQ
ANIIKLPNVSASIPQLKECIKELQSQGYKLPDYPEEPKDDKEKDIQARYAKVCGSAVNPV
LREGNSDRRAAVPVKEYAFRYPHSMGKWEPDSKTHVSCMTDGDFYSHEKSVCIEKACDAR
IELVATDGSVKVLKEKVPLQAGEVLDASFMNCKALCSFFEEQIEDAKARGVLFSLHLKAT
MMKISDPIMFGHCVKVYFKDVFAKYKETFAKLGVNANNGLGDVYKKIASLPAEEKAAIEA
DIMATYEKRGQMAMVDSHRGITNLHVPSDIIIDNSMPTAIRGGKMWNVDDKEEDFKATI
PDRGYGLVFKEVVDFCRKNGAFDPKTMGACPNVGLMAQKAEEYGSHPTTFEAPCGGIIRI
VANGTNKVLMGHHVSAGDIWRACFTKDAPIKDWCKLAVARCRANNFPNNDKPCKAIFWLD
PARAHDVVLMQKVRSYLPEFDTAGLDIEIMSPVEAMRVTCQRAKQGLNTITVTGNVLRDY
LTDLFPIMELGTSAKMLSIVPMLAGGGMYETGAGGSAPKHVQQMQKEGHLRWDSLGEYLA
LACSIADLAEDTKNGKAKALAAALDKAVGTFLVANKNPSRKVKEIDNRGSHYWLARFWAE
ELAKQEEEAELREVFTAAAKEFAEKEESVLQDLIACQGKPMDIGGYYRLDKAKADAAMNP
SETLNAIIARLEGAKKSSAKKATKITYTYTDEAPMLATHAFYPIIKSFCHHAGVPVELKD
ISVAGRVIANFSEYLTEEQRMNDILSELGQ
```

**Ortholog(s) specific to CCMP1937**

**Orthogroup number: OG0081376**
**Best-matched Uniprot annotation of orthologs (Accession numbers):**
**Pyruvate dehydrogenase kinase (P91622)**

**(Mascot score: 849)**

```
>Cluster-5567.50671
MAMHCSSRLQLASCASRVFHGTRRLASAVAPRGRARPDLRCPHDKRNFNWSSRRLFDEPA
AQPALGATIARSSRCSSSASTGTPEGEFTPGAYRIVNRVAVHRGIGLTENKFAQLHVGES
IDVTEVIHLEEEQRIRARLKEPDGWISLVDTSDGFQWAIRDLDPFDVGAWAACPEVYGHA
HSRPARQAFEPSQTVWHGDAVAPHGWGVEMVSHAGLKTPSGLNPNQDAFSTTLTSNDWII
CVVCDGHGLHGEVVAERVASALPLILVQLLLAMGPRAALSAAFAETQAHLERSLAREQFH
SGATVATCLVHKSRPECWFAHCGDSRTVLGDFASGQAVVCTQDHNPGDAAEFQRLQLAGA
KVLTKEYEGGEIVGRVFVPGTDAPGLAVSRALGDGCLKTYGVTAEPDVQDVSKAWASCRA
PGVLIASDGLWNVIDPEDAVASLARRRRMHLSLRDGAKALCQRAQETMTNLKLDQAFSYC
DDVTVALVAPPAILHVDHDDFVQSEIHRFAAKKPHRCSLQEILGMPDVEHLARFIHDEVP
VRYAERIRAIELLPGWEQNADLKEVYQRHVQTFRDIRLIQKAESKRSSLSDPLTLVEFDG
MIRNAAIKGQDVQLLVARAMYTLRLERPEVFTTEFVDRWLDDFLLNYIGTETLLMQYLAR
LEGRPNGIIDPRCDVAAVCRDVASTVQDVCMDLHQRRPQVIVESFSAVEEDQRAPSFSYI
PMFLKYVMMEILKNSCRATLVTCPYEKLPKRPTSVAVCADEHQVAIRVSDRGRGIPFHVG
NNIWSYMYSTRHKAGVGFLEGATPLAGYGIGLPLSRLYVRYLGGSLGVVSWPGLGCDVHL
SLPRLSSEQVEVVPDQDN
```

**Ortholog(s) specific to K1**

**Orthogroup number: OG0038250**
**Best-matched Uniprot annotation of orthologs (Accession numbers):**
**Fucoxanthin-chlorophyll a-c binding protein, chloroplastic (Q39709)**

```
>Cluster-24592.102021
MAQGCAFSCVDCHNQFLRLSFLSSLTMQKVAILLGCLVCTGYGKETKKALKPSDSLAKLL
LANRREAAFNSAGQAPLASSRPRSQGSMMATSRSLPWMESPAHLEGMIGNAGFDPLGIST
PENVRWMREAELKHGRMCMLAWLGYVAVDVGIKFPGERYEVLTSFNAHDATVEKELFLLL
LWVGTFETIGFNQILDMMLDPTNPRQAGDYGFDPMGIITPENEEWYKTAELTHARLAMLA
F
```

**(Mascot score: 370)**

**Orthogroup number: OG0038286**
**Best-matched Uniprot annotation of orthologs (Accession numbers):**
**Fucoxanthin-chlorophyll a-c binding protein B, chloroplastic (Q40296)**

**(Mascot score: 777)**

```
>Cluster-24592.122404
MDSRAYLAPSWLFVSSHTVHPLPPTMQKAAFVLASLVCVGQAFTPTPQQFRPVQQRSAGQ
PSASRQSVAMADSASGLSWWKPGVGNTWDPLGLAKSPEKFERLRYVEVKHGRISMLAVLG
HIVAASGARWPGQLYGGLKFADVKGSGFKALSQLSPQDYAVIVAFVAFLELRVMKEQVKG
EFPGDLRNGLFKDGWDLLTEKEKASKINKELNNGRAAMMGIFGLMVHEMLNGKPYVINEL
LGMGQPY
```

**(Mascot score: 409)**

```
>Cluster-24592.47428
MTASNTFTMKNFALVLACLVCLGQSFTPTVQQLRPSQHGPAAGRSSSKQLITMANSAPGL
SWYKAGIGNTWDPLGLAKTPEKFERLRYVEVKHGRIAMLAVLGHIVTATGYRWPGELYGH
LKFSDVKGSGFKALSQLAPQDYAVILAFVAFLELRVMKEQVKGEFPGDLRNGLFKEGWDV
LSEKEQVSKINKELNNGRAAMMGIFGLMVHEMLNGKPYVINELLGWGQPY
```

**(Mascot score: 325)**

```
>Cluster-24592.64372
MVASPHTVPSFLPTMQKVAIALASLACAGQAFTPTPQQFRPSQQRSAAQPSASRQSVAMA
DSVPGLSWWKPGVANTWDPLNLAATPEKFERLRYVEVKHGRISMLAVLGHIVAASGYRWP
GGQFYGGLKMADVKGSGFKALSQLSPQDYAVILAFVGFLELRVMKEQVKGEFPGDLRNGL
FKEGWDLLTEKEKASKINKELNNGRAAMMGIFGLMVHEMLNGKPYVINELLGMGQPY
```

**(Mascot score: 306)**

```
>Cluster-24592.96057
MSQLDPKDYAVILAFVAFLELRVMKEQVKGEFPGDLRNGLFKDGWDVLSEKEKASKINKE
LNNGRAAMMGILGLMVHEMLNGKPYVINELLGMGQ
```

**Orthogroup number: OG0019834**

**Best-matched Uniprot annotation of orthologs (Accession numbers):**

**Fucoxanthin-chlorophyll a-c binding protein F, chloroplastic (Q40300)**

**(Mascot score: 877)**

```
>Cluster-24592.21292
MVAACVSGLLNYSTSICNLIMRAQVLVFFCLACAGQARRVQMPKEHTQGAAVKELLRALK
PEAGFQAQMTQPTRIGELAARKASAPTMYDLTGEIGATGPLLPYWDPLGLAKDATPEQFR
RWRAVELKHGRIAMVATLGYAVQEVLRWPGYLSPSAGVKFADIPNGIRGLAAVPPLGLAQ
ILLAIGLMEVATWRYYEGPWPGSVPEGKEPGDVAGDLWVRYTDPEEKKYKLNIELNNGRA
AMMGSLGMLMHDHITGSWIPPGFK
```

**Orthogroup number: OG0014087**

**Best-matched Uniprot annotation of orthologs (Accession numbers):**

**Dual specificity mitogen-activated protein kinase 4 (A0A5C6NSI7)**

**(Mascot score: 454; Peptide score: 150.99)**

```
>Cluster-24592.69574
MGIEVLGVVVASLSGDPGHSMQEDSYSIIIQRNAEMEHMNVDLEDVGNKDDGGCARREET
MPLMDHVHVEEVIDREMQDNKGDAIDVRLYRLRMGAIMACAFGMVALATGIFAYHNSMSS
SRRPQDSMRFFLSNGQCKRPAGWCMSNPCFTGNYSLYDCDGDGQLDPFCWPALAQPGDRL
CGCLQSSNGCIDTKLFNFLCPAPTTAAPTPAPTPAPTPAPTPAPTPAPTTTAAEPTMTLP
TPYIPVVAPKESILAAWGDDIKVDYCVPKGHLVGTFNITGKMIDVKMPEDGCITAKAPKV
VNDPIYEGYQLIKYRHPKSVDLSSSDMKEVKSPYPGIMKEWLKSVGAKVDPGDAVARIFV
NGDVKTIAYTDGPSGVVVDRAPFLPSDPVAAGTDLIVVALGIFVKWEADVGEHVKKDDLI
CSMTQGYIVKPDGIPADTAANDEILGWVAANSKKLFPKSTIPLLASKNGSVVQRSDEEPG
DIIGINRKTPGTALILSHLPPVPVPASYISRYNSTPPNVTKAPDGAGVTPKWYSSDGSKY
LYTGAWLKLKAWKVESGDQVKSGEMVATAENLEGVSSNLTTRYSGYIVNKQYINPGDFIG
PSDELVLVGNYLAPAAGSSFPWWVLLLPLLCLLCLIPLCCKSEPPPPPPPPPPPPPPVVV
EPAPAPVVVAPPPPKPEPKPEPKPEPKPEPKPEPVVVPPPPPPGFPIYFDGKEYRCEYHP
LGIKFWQKAPIKVEEYNFNSYGKTLGITKGMTLTKIKDTEIRPDQKFKDVHHLLLEAMAP
LPVWPLHMGFKTPGGELKEYWFNEHPIGIEFTRQAPIKIDTVKSGSLAAEVGAQPGWTIV
KLGDSDISGDHNFSHVSKLLKDGLHNLPERGKAQMGTYADNLGAGSHSGGGVTEAPRPST
EAPAGFK
```

**(Mascot score: 804; Peptide score: 128.21)**

```
>Cluster-24592.107035
MTMRIAILVFACLALTDAQRVRLTGEPREATTQALSALLQTRKPTAGFQPSGNVLGNTPR
SPSSKLAERPAQPQMVDLTGQLGATGPLLPYWDPLGLAKDATPEQFRRWRAVELKHGRIA
MAATLGYFVQEVLRWPGYLSPSAGIKFSDIPNGIRGLAAVPPLGLAQILLGIGLMEVATW
RYYEGPWPGSVPEGKEPGDVAGELWVRYSDPEEKKTKLNIELNNGRAAMMGSLGMLMHDH
LTGSWIPPGFK
```

**Orthogroup number: OG0019848**
**Best-matched Uniprot annotation of orthologs (Accession numbers):**
**CAAX amino terminal protease family protein (M2X807)**

**(Mascot score: 268)**

```
>Cluster-24592.117554
MSWLLKIDRVTFISFFTMRLALLLVCLACVGHGRRVYTSPGDSHSSALAGRQKTSKGIDV
DSRRLGAPIASRSLAALLRAGSPAAGITASSRPTLPHLPSRTAATPPRSLRSVLGLRGGR
STGGEEEWPEEELEDDGMAIDVERGSSDVASEQEVVLDENDATKAEESDSSPSEDSGDAV
YYKGGASGELTEENVLPTLVQTPILIAAYLLHLTWVSKNQLPLGRRSLPYENIVGAVAVS
NYFVSQIRKWWLRRQQGLEATPTTVPWGKVHNGPFSLLLTTLMCMGAHWLSNYIGIAFDY
FLLFLENRGVPMTWGQAYAMQQLFSHLAYVAMAVHAMGSRLKPFFPPPFGKGEWIRVRWH
TNWLGWVVGGFYASTLACNSLTASCHALYNYLVKNAGLPPAPTDVQPLTETVVNQLSRPG
GGDIAAFLVGLLAPCVSAPIFEEVLYRGFLLPTLHRFMPLGAALPLQGLLFGLHHRAIPT
LLPLSLLGWLWGWFYVKTGNLVIPMLMHAMWNSRSFVNSLLEKAY
```

Peptide ion identified by Mass Spectrometry

**Amino acid residue labeled with the isotopic tag**

**Table S5.** Uniprot annotations and amino acid sequences of STX-producing-species-specific surface orthologs identified.

**Orthogroup number: OG0034291**

**Best-matched Uniprot annotation of orthologs (Accession numbers):**
**F-type H+-transporting ATPase subunit beta (A6BM09, Q06J29)**
**Ortholog(s) of CCMP113**

**(Mascot score: 2,553)**

```
>Cluster-16584.51079
MKCKVFDFEKFMMTQIRLVQRYVFLPSVYDALLVMRPSCNFRDGTVVKNCVANFGQYFVE
LPFNDSYLFGLIHLIPGDYKSTLHCFYSSMCREVCNVRDIVLGFASVFLFVKSYANCLVA
ELSQLCYGGVLRAVALGSTDGLCTWRCTFLMVLQPVIVPVGRIALGRIFNVVGSVIDRYM
ELSLSSQFNVSVPIDNGLFVESCEDLTYTLGYPDAILTLVHTKALDLNLISLWNTMVVST
ISKSWIYYIGCLYQLVISQVCRLHQNLKYGTHESVIFEEIDRSFNALSRACESLFYNTDT
LFALVKPIHKTPVAIMTLSIHLTLFETGIKVVDLLTPYKKGGKIGLFGGAGVGKTVVIME
LIRNLAVEHGGLSLFAGVGERTREGNDLYCEMQDSGIISLTLRQGKSQSSVCYPCLMYQP
LFAAAWSQVVLVFGQMNETPGSRMRVTHASLAAAEYFREAFCQDVLIFVDNVFRFLQAGS
EVSTLLGRMPSAVGYQPTLSTEMASFQERIVATTAGSITSIQAIYVPADDLTDPAPVVIF
GHLDAVTVLSRALASKGIYPAVDALNSTSKMLDPSYVKQEHFCVATDVKQMLQRYKELQD
VIAILGLEELSDQDRIVVDRARKVERFLSQPFFVAEVFTRIQGRYVSLNDTIIGFSQIVQ
GELDEVSEGGFYLKGALSDVVHDNDA
```

**Ortholog(s) of CCMP1888**

**(Mascot score: 1,414)**

```
>Cluster-6994.52323
MKCKVFDFEKFMMTQIRLVQRYVFLPSVYDALLVMRPSCNFRDGTVVKNCVANFGQYFVE
LPFNDSYLFGLIHLIPGDYKSTLHCFYSSMCREVCNVRDIVLGFASVFLFVKSYANCLVA
ELSQLCYGGVLRAVALGSTDGLCTWRCTFLMVLQPVIVPVGRIALGRIFNVVGSVIDRYM
ELSLSSQFNVSVPIDNGLFVESCEDLTYTLGYPDAILTLVHTKALDLNLISLWNTMVVST
ISKSWIYYIGCLYQLVISQVCRLHQNLKYGTHESVIFEEIDRSFNALSRACESLFYNTDT
LFALVKPIHKTPVAIMTLSIHLTLFETGIKVVDLLTPYKKGGKIGLFGGAGVGKTVVIME
LIRNLAVEHGGLSLFAGVGERTREGNDLYCEMQDSGIISLTLRQGKSQSSVCYPCLMYQP
LFAAAWSQVVLVFGQMNETPGSRMRVTHASLAAAEYFREAFCQDVLIFVDNVFRFLQAGS
EVSTLLGRMPSAVGYQPTLSTEMASFQERIVATTAGSITSIQAIYVPADDLTDPAPVVIF
GHLDAVTVLSRALASKGIYPAVDALNSTSKMLDPSYVKQEHFCVATDVKQMLQRYKELQD
VIAILGLEELSDQDRIVVDRARKVERFLSQPFFVAEVFTRIQGRYVSLNDTIIGFSQIVQ
GELDEVSEGGFYLKGALSDVVHDNDA
```

**Ortholog(s) of ATCI03**

**(Mascot score: 2,425)**

```
>Cluster-15238.40798
MRNHGSVCSIVGPVIDVNMKCLVFNFDKFMITQIARPSGFVVESNVFLPSVYDAILVIRP
SCNFRDGTVVNNCIVNYGQYLCELPFNDSSLLGLIHLLPVQVKYLTIHSFWNSGSSDTHS
AMCKEACEMNIRDILFGFASVFLFIKSYANCLVAELSQLCYGGVLRAVALGPTDGLCTWG
CSFLMVLQPVIVPVGRIALGRIFNVVGSGIDRYMELSLSCQFNTAVPIDLGLFVESHPEL
TYALSYPDTILTLAHTKALDFSLVSHWNTILSSNTISSAWVYYIAYLYQLVMNHVHLNVK
DRTQKIKISEIDCSVDALSRACESLFYNTDTLFALGKPIHQTPVAIMTLSIHLTLFETGI
KVVDLLTPYKKGGKIGLFGGAGVGKTVVIMELIRNLAVEHGGLSLFAGVGERTREGNDLY
CEMQDSGIISLTLRYPDPTHPRLMYQPLFAANQSQVVLVFGQMNETPGSRMRVTHASLAA
AEYFRDAFGQDVLIFVDNVFRFLQAGSEVSTLLGRMPSAVGYQPTLSTEMGSFQERIVAT
NAGSITSIQAIYVPADDLTDPAPVVIFGHLDAVTVLSRALASKGIYPAVDPLNSTSKMLD
PSYVKQEHFCVATDVKQMLQRYKELQDVIAILGLEELADQDRIVVDRARKVERFLSQPFF
VAEIFTRIQGRYVSLLDTIRGFSQIIKGELDQVSEGGFYLKGALSDVTFGSECIRHVISS
NTNVPNT
```

**Ortholog(s) of CCMP1937**

**(Mascot score: 1,463)**

```
>Cluster-5567.44538
MITQIRPVIADQQLHPLVRDVFFPSVYDALLVITSQCNFRDGTVIYNVGSNQAQYFAELP
FNDAYSVALIHLCCAVFDSRSPVARGCTPCGVHEYIGMTQISSEIWDMNARDVVLGFAAL
FLLVKSYANCLVAELSQLWQGGVMRAIALGSTDGLSCFSCTFLLTFQPVIVPVGRITLGR
ILNVVGSSIDPYIHLSLSSQFGPYHLVSAELFVESSLESSSYATTFISEGCLALDHSLIS
LYTTMVVSTISFPWIYYLACLCRAMVRCMAVCVPPESVGLLDMLESCRSTDGHYKTIDRS
VEVIQHWCNINFYHADTLFASIKPIHGTPLAINELRICVILFETGIKVVDLLTPYKCGGK
IGLFGGAGVGKTVVIMELIRNLAVEHSGLSLFAGVGERTREGNDLYCEMQDSGIIKISLS
```

```
IPKEQYSLNPEGSSYSVVTRLSQVVCVFGQMNETPGSRMRVTHASISMAEYFRDAFSQDV
LIFVDNVFRFLQAGSEVSTLLGRMPSAVGYQPTLSTEMGSFQERICARLTGSITSIQAIY
VPADDLTDPAPVLIFGHLDATTVLSRALASKGIYPAVDPFNSTSKMLCPECVKQEHFCVA
SDVKQMLQRYKELQDLIAILGLEELSDQDRIVVDRARKIERFLSQPFFVAEVFTRISGRY
VSLNDTIRGFTKIVTGELDAQSEGGFYLKGAITDVM
```

Peptide ion identified in Mass Spectrometry

**Amino acid labeled with isotopic tag**
